# Cost-Effective Potentiometric Platforms Modified with Multi-Walled Carbon Nanotubes (MWCNTs) and Based on Imprinted Receptors for Fluvoxamine Assessment

**DOI:** 10.3390/polym12030673

**Published:** 2020-03-17

**Authors:** Heba M. Hashem, Saad S. M. Hassan, Ayman H. Kamel, Abd El-Galil E. Amr, E. M. AbdelBary

**Affiliations:** 1 Chemistry Department, Faculty of Science, Ain Shams University, 11566 Abbasia, Cairo, Egypt; hebahashem426@yahoo.com; 2 Pharmaceutical Chemistry Department, Drug Exploration & Development Chair (DEDC), College of Pharmacy, King Saud University, Riyadh 11451, Saudi Arabia; 3 Applied Organic Chemistry Department, National Research Center, 12622 Dokki, Giza, Egypt; 4 Chemistry Department, Faculty of Science, Mansoura University, 35516 Mansoura, Egypt; ebary301@yahoo.com

**Keywords:** molecular imprinting polymers (MIP), screen-printed, solid contact, ion-selective electrodes (ISEs), fluvoxamine, MWCNTs

## Abstract

A simple, efficient and reliable analytical method was developed and used for the determination of the fluvoxamine drug (FLV) in pharmaceutical preparations and biological fluids. The method is based on the cost-effective screen-printed platform for the potential transduction of the drug. Host-tailored molecular imprinting polymer (MIP) was integrated with the potentiometric platform as a recognition receptor, in which FLV, acrylamide (AAm), ethylene glycol dimethacrylate (EGDMA) and acetonitrile were used as a template, functional monomer, cross-linker, and solvent, respectively. MIP particles were dispersed in plasticized poly (vinyl chloride) (PVC) and the membrane was drop-casted on carbon screen-printed electrode. The MIP, in addition to non-imprinted polymers (NIP), was characterized and the binding experiment revealed high affinity and adsorption capacity of MIP towards FLV. The proposed sensor displayed near-Nernstian cationic slope of 55.0 ± 0.8 mV/decade (*r^2^* = 0.999) with a low detection limit of 4.8 × 10^−6^ mol/L over a wide pH range (3.0–8.5). The electrochemical features of the proposed sensors including electrochemical impedance spectroscopy (EIS) and chronopotentiometry measurements (CP) in the presence of multi-walled carbon nanotubes (MWCNTs) as a solid contact transducer were also investigated. The applications of the proposed sensor for the determination of FLV in different dosage forms with recovery values (98.8%–101.9%) and (97.4%–101.1%), respectively compared with the reference HPLC method with acceptedFandt-student tests values at the 95% confidence level.

## 1. Introduction

Depression has a massive impact as a disability, which is a common and invalidating mental illness affecting approximately 2.5% of the general population as shown in the last guidelines update of the world health organization (WHO) in 2018 [1,2]. It harms a person’s behavior and their social consequences in terms of reduced employment and psychosocial impairment. Postpartum depression (PPD) is one of the major depressive disorders that occur within the first month after childbirth [3,4]. Research shows that more severe forms of depression are associated with specific changes to some hormones besides the chemical message system.

**Figure 1:**
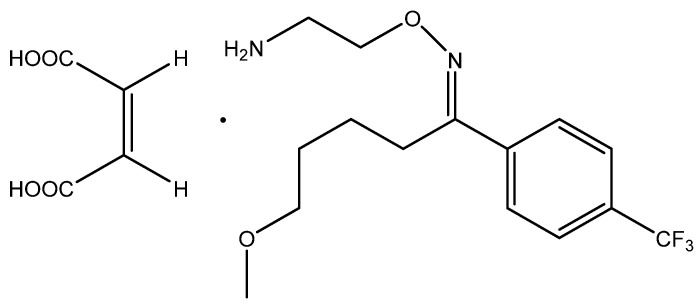
Of fluvoxamine maleate.

Fluvoxamine maleate (FLV) (5-methoxy-4-(trifluoromethyl) valerophenone (E)-O-(2- aminoethyl) oxime maleate) (Figure 1) has high efficacy in the treatment of different types of depression and increases the synaptic serotonin by selective inhibition of serotonin uptake into presynaptic neurons [5]. Also, it is considered as an effective antidepressant agent instead of tricyclic agents due to its minor effects on the nor-adrenaline and dopamine re-uptake with favorable tolerability profile [6,7]. Therefore, it is crucial for the determination of FLV in pharmaceuticals and biological fluids.

Many different analytical techniques have been developed and reported for FLV quantification, such as high performance liquid chromatography (HPLC)coupled with either fluorescence [8,9] or ultra-violet (UV) detectors [10,11,12,13,14,15,16], gas chromatography/mass detection (GC/MS) [17], gas chromatography/flame ionization detection (FID) [18], capillary electrophoresis [19,20,21], fluorimetry [22], flow-injection chemiluminescence (FI-CL) [23,24], electrochemical methods [25,26,27] and spectrophotometry [28,29,30]. Most of these techniques required prior derivatization or extraction steps and involve using expensive equipment and chemicals. Consequently, it is vital to design a fast, simple, sensitive, selective and robust method for the determination of FLV concentrations. Ion-selective electrodes (ISE)-based MIPs have high sensitivity, high selectivity, low cost and ease of miniaturization and automation [31,32] that have been used for the determination of pharmaceutical drugs and insecticides [33,34,35,36,37].

According to suitable therapeutic drug monitoring purposes, looking forward to predetermined recognition ability, relatively easy, low cost of preparation and mechanical and chemical stability, molecular imprinted polymers (MIPs) have a wide attention and have been used for various applications, such as catalysis [38], quantification of toxins in food matrices [36,39], solid-phase extraction (SPE) [40], drug delivery [41,42], biological antibodies and receptors systems [43,44] and finally chemical sensors [45,46]. Exceptional properties of MIPs based on their synthesis procedure and their high selectivity against a specific drug molecule returns to the presence of specific recognition sites within their cavities [47]. The screen-printed solid-contact electrode was well established for the mass production of disposable electrochemical sensors and for avoiding the disadvantages of conventional ISE [32,48]. It could be a promising technique for the determination of organic ions. To the best of our findings, only a few cited papers for screen-printed potentiometric sensors based on MIPs were found [32,34,36].

In this work, the establishment of the cost-effective potentiometric sensor by using a carbon screen-printed electrode modified with multi-walled carbon nanotubes (MWCNTs) as a solid contact material for the determination of FLV drug in different samples. Artificial MIP receptors used as a selective recognition part of the proposed sensors. The sensitivity, selectivity, and applicability of the proposed sensors were investigated. The potential drift and the double-layer capacitance of the sensors were tested using chronopotentiometry and electrochemical impedance spectroscopy. The applicability of the sensors towards real samples was checked in determining FLV in different pharmaceutical formulations.

## 2. Materials and Methods 

### 2.1. Reagents and Chemicals

All reagents were of analytical grade and doubly distilled water was used as received. High molecular weight poly (vinyl chloride) (PVC), dioctyl phthalate (DOP), sodium tetraphenylborate (TPB-), acrylamide (AAm), benzoyl peroxide (BPO) and ethylene glycol dimethacrylate (EGDMA 98%) were obtained from Sigma Aldrich (St. Louis, MO, USA). MWCNTs were purchased from (EPRI, Cairo, Egypt). Fluvoxamine maleate drug was obtained from Pharaonia Pharmaceuticals (Alexandria, Egypt). Tetrahydrofuran (THF) and acetonitrile were obtained from Fluka AG (Buchs, Switzerland). Tetrahydrofuran (THF) was freshly distilled prior to use.

A stock solution of FLV (1.0 × 10^−2^ mol/L) was prepared by dissolving the definite weight of pure FLV in 100 mL double distilled water and (10 mmol/L) phosphate buffer solution at pH 6.0 was prepared for calibration measurement. The working solutions (1.0 × 10^−2^ − 1.0 × 10^−7^ mol/L) were prepared with accurate dilutions and stored in brown bottles at 4 °C.

### 2.2. Apparatus

Potential measurements were recorded at 25 ± 1 °C with a bench pH/mV meter (A Jenway™ 3510, Staffordshire, UK) using a fluvoxamine PVC membrane sensor in conjunction with Jenway™ Ag/AgCl double junction reference electrode filled in the outer compartment with 1M CH_3_COOLi. When necessary, pH values were controlled by means of a combined glass pH electrode (Jenway™3505). For the characterization of the polymer, infrared spectra were recorded on Attenuated Total Reflection (ATR) Fourier transform spectrometer (Thermo-Fisher Scientific iS10, Waltham, MA, USA). The surface morphology of the synthesized polymer beads was analyzed using a scanning electron microscope (SEM) (JEOL JSM 6510lV, Osaka, Japan). A Shimadzu UV/VIS spectrophotometer (Shimadzu UV-1601 PC, Kyoto, Japan) was used for the absorbance measurements of the binding analysis. High-performance liquid chromatography (HPLC) coupled with UV/VIS detector (Series 200 Pump, Perkin Elmer, Waltham, MA, USA) was used for the measurements of the reference method.

### 2.3. Synthesis of Host-Tailored Receptors

MIP was prepared by mixing 1.0 mmol of FLV as a template, 3.0 mmol of AAm as functional monomer, 3.0 mmol of EGDMA as a cross-linker and 80 mg of benzoyl peroxide as an initiator. All the previous components were dissolved in 15 mL of acetonitrile and were de-gassed with N_2_ bubbling for 5 min, followed by good sealing under the nitrogen atmosphere. Then, the mixture was placed in a thermo-stated (paraffin wax) bath at 70–80 °C for 18 h. Non-imprinted polymer (NIP) was prepared similarly but without the addition of the template. The resulting powders were dried and collected. Washing MIP from undesired species and elution of the drug molecule from it occurred several times with absolute ethanol in a Soxhlet extractor for 48h. MIP was left untilcomplete dryness at ambient temperature before using it as an ionophore in sensor membranes.

### 2.4. Fabrication of the Sensors and EMF Measurements

The design of the ceramic screen-printed electrode (SPE) is designed to contain one screen made from carbon and printed on an alumina substrate of 0.1 mm thickness and 35 mm length. The screen of carbon was of 2 mm width. Ten µL of MWCNTs (0.1 g/25 mL THF) was deposited by drop-casting onto the carbon sensing area. After that, the solution was left to dry for 3 min. The recognition membrane was prepared from 68.0 mg of PVC powder, 118.0 mg of DOP as a plasticizer, 2.0 mg of additive (TPB^−^) and 12.0 mg of MIP. All previous species were dispersed in 3.0 mL THF with vigorous stirring until complete homogeneity. After that, 10-µL of MWCNTs (0.1g/25 mL THF) was loaded on the orifice of the carbon screen-printed electrode and left it until complete dryness. Ten µL of the previous dispersion cocktail was drop-casted over the previously deposited MWCNTs layer and then left to dry. Conditioning of the proposed sensor was done by soaking it in 1.0 × 10^−2^ mol/L FLV solution for 2 h before using. The storage of the sensor was done in the previous solutions when it is not in use. EMF values were measured for various concentrations after potential stabilization ±2 mV and a plot between the resulting EMF values and logarithm [FLV] concentration was obtained.

### 2.5. Analytical Applications

FLV is administered by the oral dosage of pharmaceutical formulations which have labeled commercially as Fluxamine tablets (50,100 mg tablet^−1^) (Pharaonia pharmaceuticals “Pharo Pharma”, Egypt) and Faverin^®^ tablets (Abbott Healthcare SAS) contained 50 and 100 mg FLV per tablet. A definite amount of the mixed finely powdered 10 tablets, equivalent to one tablet, was transferred into a 100 mL volumetric flask then completed to the mark with double distilled water. The content of the flask was sonicated for 45 min to ensure complete dissolution. From the clear supernatant, the different concentrations were prepared. After that, the potential measurements were recorded and compared with the calibration of the pure drug under similar conditions.

## 3. Results and Discussions

### 3.1. Characterization of the MIP Particles

#### 3.1.1. FT-IR Analysis

**Figure 2:**
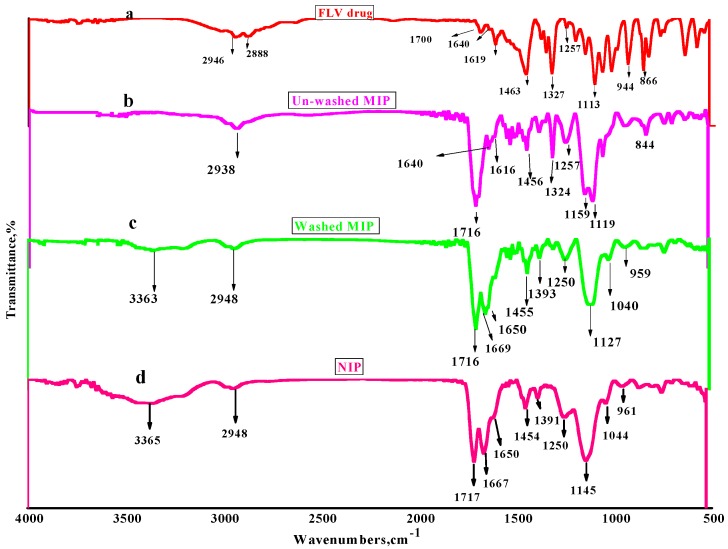
Fourier transform Infrared (FT-IR) spectra of fluvoxamine (FLV) (**a**), non-washed MIP (**b**), washed MIP (**c**) and NIP beads (**d**).

The investigation of the imprinting process through imprinting of cross-linked AAm monomer with FLV drug as a template can be illustrated in FT-IR spectra as following. In Figure 2a, the FLV spectrum showed stretching C–H and acidic O–H peaks at 2946 and 2888 cm^−1^. The last peak attributed to the presence of O–H of the carboxylic group in maleic acid and the broadband confirms the presence of hydrogen bond. Weak peaks appeared at 1700, 1640, and 1619 cm^−1^ due to the presence of acidic –C=O stretching (COOH), C=N and N–H bending of secondary amine group, respectively. Besides, strong and sharp peaks at 1463 and 1327 cm^−1^ attributed to C–H bend and C–F stretching, respectively. Besides, all assignable peaks appeared at 1257 to 1113 cm^−1^ referred to C–O stretches of ether. Strong and sharp peaks of para-substituted aryl group assigned at 950 to 650 cm^−1^. As can be clear in Figure 2b, un-washed MIP is characteristic with assignable peaks shown at 1640 and 1616 cm^−1^ that referred to C=N and N–H bending of secondary amine. Also, C–F stretches and para-substituted aryl groups assigned at 1324 and 844cm^−1^, respectively, which found in the FLV spectrum. Strong peaks appeared at 1119 cm^−1^ attributed to ether C–O stretching of FLV, which confirms the progress of the imprinting process between cross-linked AAm and FLV. All previous peaks completely disappeared in Figure 2c,d, which confirm completely disappearance of FLV. But three spectrums of unwashed, washed MIP and NIP have strong peaks that showed at 1716 and 1257–1119 cm^−1^ were attributed to kenotic–C=O and C–O stretches, respectively refers to presence of EGDMA cross-linker and AAm monomer. Strong peaks showed at 1669 and 1650 cm^−1^ attributed to N–H bending of amide CONH_2_ group. These peaks are completely disappeared in Figure 2b, which refers to the non-washed MIP. This refers to the contribution of the NH_2_ group in the imprinting of the FLV molecule. Also, a medium peak that appeared at 1250 cm^−1^ in both washed MIP and NIP polymers can be attributed to the C–N stretch of aliphatic amine. Finally, all spectrums demonstrated the imprinting behavior of FLV with cross-linked AAm monomer and confirm complete removal of FLV from MIP after washing to be adequate for use as an ionophore in the proposed sensor.

#### 3.1.2. SEM Analysis

**Figure 3:**
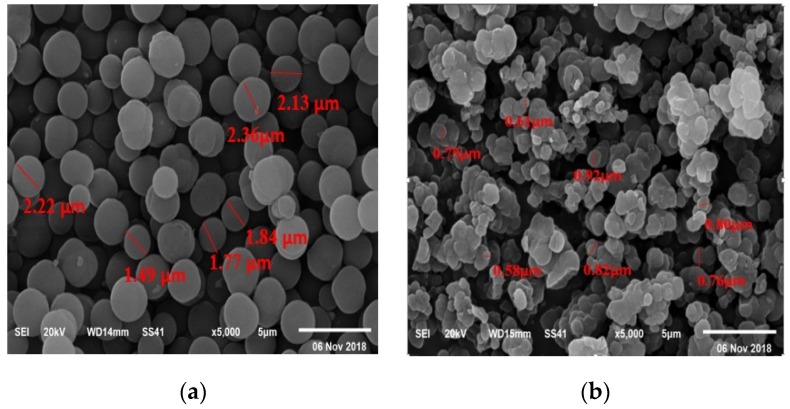
SEM images of (**a**) NIP and (**b**) washed MIP beads.

The morphologies of both NIP and MIP after washing were investigated and presented in the SEM micrographs in Figure 3. The surface morphology of NIP beads referred to spherical, smooth and uniform shapes with a size ranged between 1 to 2 µm diameters (Figure 3a). This can be attributed to the absence of specific binding sites in the polymers. Figure 3b shows MIP surface morphology that revealed the distorted beads with an irregular shape with beads averages diameter ranged between 0.5 to 0.9 µm. This can be attributed to the imprinting process. These morphology differences can confirm the high efficiency of MIP beads to adsorb and uptake FLV more than NIP beads.

#### 3.1.3. Binding Isotherms and Scatchard Analysis

**Figure 4:**
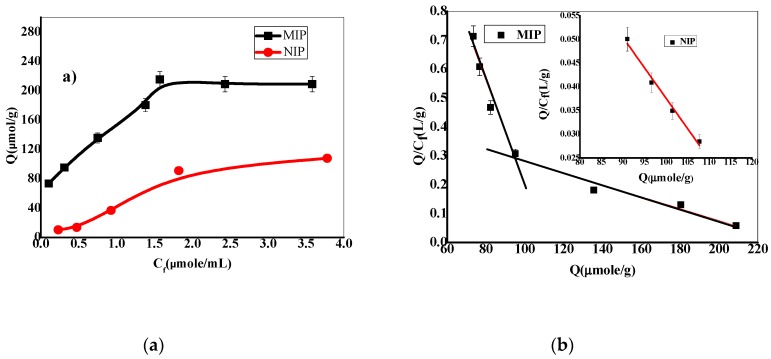
Binding isotherm (**a**) and Scatchard plot (**b**) for MIP and NIP.

The maximum binding capacities for the synthesized polymers were also evaluated. 20.0 mg of either washed MIP or NIP beads was placed in contact with 10.0 mL FLV aqueous solutions that have concentrations range from 0.25–5.0 mmol/L. The solutions were shacked with 90 rpm overnight at 25 ± 1 °C. The solid phase was separated by centrifugation (3000 rpm, 15 min.). Free FLV concentrations in the supernatant were measured by UV spectrophotometry at 250 nm using a calibration graph with FLV standard solutions [49]. The binding capacity of either MIP or NIP was calculated according to Equation (1):

(1) $$Q=\frac{\mu mol\left(FLV\;bound\right)}{g\left(MIP/NIP\right)}=\frac{\left(Ci-Cf\right)Vs\times 1000}{m\left(MIP/NIP\right)}$$

where *Q* is the binding capacity (μmol/g), *C_i_*, *C_f_*, *V_s_* and *m* are the initial FLV concentration (μmol/mL), the final FLV concentration (μmol/mL), the volume of tested solution (mL) and the mass of the dried polymer (g), respectively. As shown in Figure 4a, the adsorption capacity of MIP beads adsorption capacity is much higher than in NIP beads. This indicates a stronger affinity of MIP towards FLV than NIP.

Scatchard analysis was carried out and shown in Figure 4b. The Scatchard binding characteristics were evaluated according to the following Equation (2) [33]:

(2) $$\frac{Q}{C_{f}}=\frac{\mathrm{Qmax}-Q}{K_{d}}$$

where *Q* is the binding capacity, *C_f_*, *Q_max_,* and *K_d_* are the free analytical concentration at equilibrium (μmol/mL), the maximum apparent binding capacity and the dissociation constant at binding sites, respectively. It can be seen that *K_d_* and *Q_max_* values were 55.3µmol/L and 485.9 µmol/g for high affinity and 110.9 µmol/L, 237.2 µmol/g for low-affinity binding sites of MIP, respectively. Two linear regression equations indicate that two different binding sites for FLV were formed in MIP, which are known as specific and non-specific binding sites from imprinting cavities [50]. In contrast, the *K_d_* and *Q_max_* values were 781.4 µmol/L and 129.4 µmol/g for NIP, respectively. This indicates a high homogeneity of the binding sites present in NIP beads and shows a lower affinity towards FLV than MIP [51].

Using Equation (3) it is possible to estimate Gibbs free energy change of FLV/MIP complex formation:

Δ*G* = *RT*/ln *K*_D_ (3)

where the *K*_D_^0^ = *K*_D_/*C*, in this case the *C* is the concentration of FLV; Therefore, the *K*_D_^0^ is the value, which is calculated for standard reference concentration of 1 M [52,53]. It was found that Δ*G*= −0.252 and −0.272 kJ/mol for FLV/MIP complex formation for both high affinity and low-affinity binding sites of MIP, respectively. Δ*G* value for NIP particles was found to be −3.45 kJ/mol for FLV/NIP complex formation. This shows that FLV/MIP complex formation is a thermodynamically favorable reaction.

### 3.2. Analytical Features of the Proposed Sensors

#### 3.2.1. Membrane Optimization

Studying the essential analytical features of the proposed sensor and its sensitivity was established according to the presence and absence of TPB^−^ as an anionic additive. From Table 1, all membranes were prepared with definite weights (68.0 and 118.0 mg) of PVC and DOP, respectively in presence of MIP or NIP (0.12 mg) was dissolved in 2.0 mL THF. The presence of TPB^−^ (2.0 mg) in the membrane of the applied sensor (I) enhanced the potentiometric response of the applied sensor, which exhibited near-Nernstian response with the cationic slope of 55.0 ± 0.8 mV/decade (*n* = 6, *r^2^* = 0.999) and lower detection limit of 4.7 × 10^−6^ mol/L. However, the absence of TPB^−^ as the sensor (III) exhibited a sub-Nernstian slope of 32.8 ± 1.4 mV/decade (*n* = 6, *r^2^* = 0.995) and higher detection limit of 1.0 × 10^−5^ mol/L, which revealed that the presence of TPB^−^ enhancing of the potentiometric response of the applied sensor. Blank (sensor II) with the absence of MIP and presence of TPB^−^ revealed the near-Nernstian slope of 55.4 ± 0.5 mV/decade (*n* = 6, *r^2^* = 0.999) and detection limit of 1.2 × 10^−5^ mol/L.

Also, the presence of MWCNT as a transducer in the proposed sensor (IV) exhibited a near-Nernstian slope of 56.2 ± 0.6 mV/decade (*n* = 6, *r^2^* = 0.999) and detection limit of 4.2 × 10^−6^ mol/L, which revealed that there is no difference between its presence and its absence in the potentiometric response. Sensor (V) was established as control with NIP beads based membrane that exhibited a sub-Nernstian slope of 29.7 ± 1.4 mV/decade (*n* = 6, *r^2^* = 0.999) and detection limit of 1.1 × 10^−5^ mol/L, which confirm the efficiency and high sensitivity of selected MIP beads for the detection and determination of FLV in different samples.

**Figure 5:**
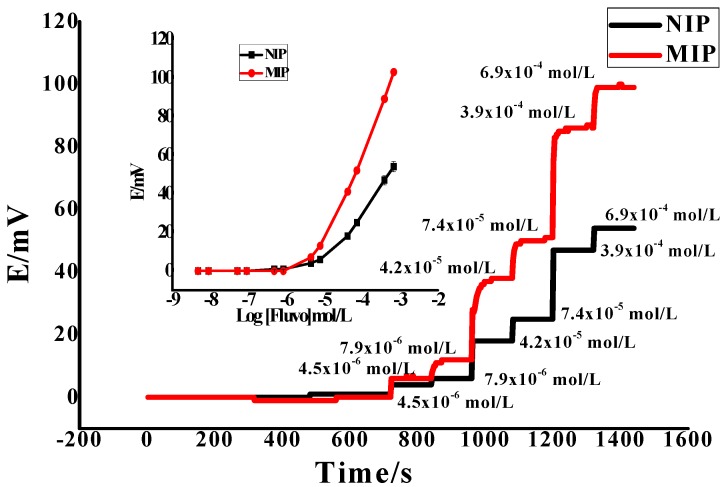
The potentiometric and time response of non-imprinted polymer (NIP) and the proposed sensor (MIP = 12, TPB^−^ = 2.0, PVC = 68 and DOP = 118 mg) in phosphate buffer (0.01 M) pH = 6 with MWCNT as a transducer.

As shown in Figure 5, the steady potential response time applied using the proposed sensor in 1.0 × 10^−7^ − 1.0 × 10^−2^ mol/L FLV solutions with a 10-fold rapid increase in concentration was about 10–20 s. This was applied by recording the potential readings at time intervals of 3 s over 2 min for each half mL from each concentration of FLV. The results confirm that the proposed sensor has high stability and it can be used as a fast and automated analytical method. The repeatability of the applied sensor was tested by using 1.0 × 10^−3^ mol/L in pH 6 over 2.5 h, which revealed no significant change in the potentiometric response. After recalibrating the applied sensor over 10 times per day, it can be seen that no significant change with the high reproducibility of cationic slope 55.0 ± 1.2 mV/decade (*n* = 6) and detection limit of 4.3 × 10^−6^ mol/L (*n* = 10).

#### 3.2.2. Method Robustness

**Figure 6:**
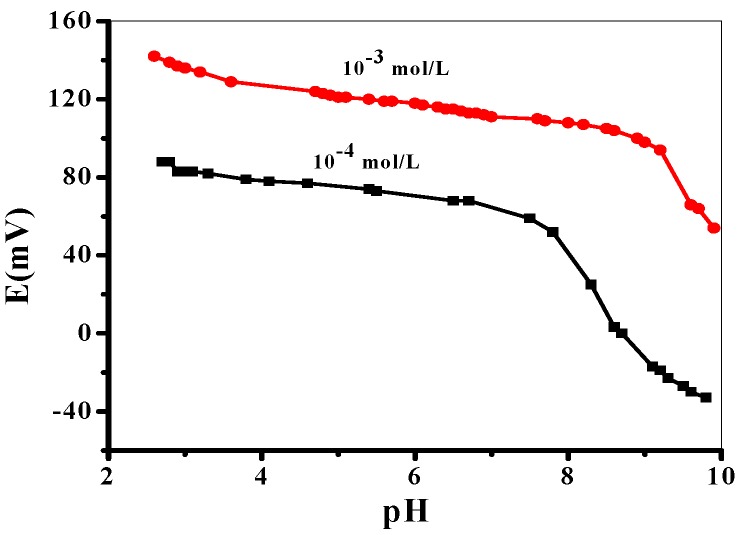
Effect of pH on the potentiometric response of the applied sensor.

The impact of pH on the potential response of the proposed sensor was examined in the pH range of 2–10. The potential readings of FLV test solutions (1.0 × 10^−4^ and 1.0 × 10^−3^ mol/L) at various pH values were recorded. The pH of the test solutions was adjusted by the addition of very small volumes of HCl and/or NaOH solutions. As can be seen in Figure 6, the potential was kept constant in the pH range of 3.0–8.5, which can be taken as the working pH range for FLV determination. Therefore, 10 mmol/L phosphate buffer at pH 6 was chosen for all subsequent measurements. At pH < 3, the higher potential response is noticed due to the interference of H^+^ ions. At pH > 8.5, the potential begins to be declined due to the formation of the free form of FLV base (*pK*a = 8.7) in the test solution, which cannot be detected by the sensor.

#### 3.2.3. Selectivity Studies

The selectivity coefficient ($\log\;K_{\mathrm{FLV},J}^{\mathrm{Pot}}$) determines the ability of an ISE to respond to an analyte against an interfering ion *j*. The selectivity coefficients of the FLV sensors towards foreign compounds were calculated using the modified separate solution method (MSSM) [54]. As shown in Table 2, the selectivity coefficient values ($\log\;K_{\mathrm{FLV},J}^{\mathrm{Pot}}$ of the proposed sensor with and without MWCNT have no distinct differences. In addition, the values revealed that no interference with FLV determination over K^+^, Na^+^, Ba^2+^, Ca^2+^, caffeine, arginine, glucose, lactose, fluxatine, Venlafaxine, ephedrine and paracetamol. In addition, the results confirm the broad suitability of the developed sensor for high selectivity determination of FLV.

### 3.3. Chronopotentiometry (CP) and Electrochemical Impedance (EI) Measurements

**Figure 7:**
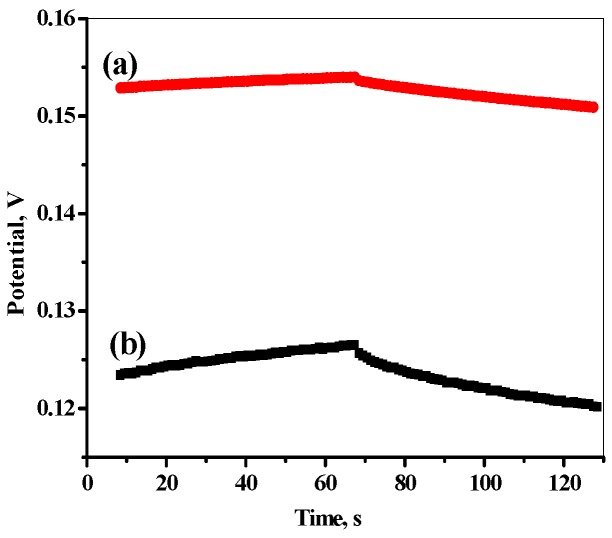
Potential stability of the applied sensor (**a**) with and (**b**) without MWCNT as a solid contact material.

Chronopotentiometry measurements (CP) of the proposed sensor-based MIP was carried by applying constant-current using one-compartment three-electrode cell using (NOVA 2.0 software; Metrohm Auto lap B.V., Utrecht, The Netherlands) combined with Pt auxiliary electrode and the reference electrode (Ag/AgCl/KCl (3 mol/L). The measurements were applied in a solution of 10^−3^ mol/L of FLV in phosphate buffer pH 6 at room temperature 25 ± 1 °C. CP was applied at current ±1nA for the 60s. Figure 7 shows chronopotentiograms (E–tcurves), which have two main properties. First, a potential-jump when changing the direction of the current and a slow potential drift at longer times. Second, the potential jump in the E-t curves can be used to estimate the total resistance (*R*_b_) of the electrode, which is controlled by the bulk resistance of the plasticized PVC-based membrane. The results of *R*_b_ and *C*_L_ in the presence and absence of MWCNTs are (0.23 ± 0.02 MΩ, 23.9 ± 1.2 µF) and (0.69 ± 0.05 MΩ, 5.07 ± 0.1 µF), respectively. The potential drift (∆*E*/∆*t***)** of the applied sensor with MWCNT (46.5 ± 1.5 µV/s) is lower than in case of its absence (197.3 ± 12.3 µV/s) that indicate too high potential stability, high capacitance and low impedance of the sensor was investigated by addition of MWCNT as a solid contact material.

**Figure 8:**
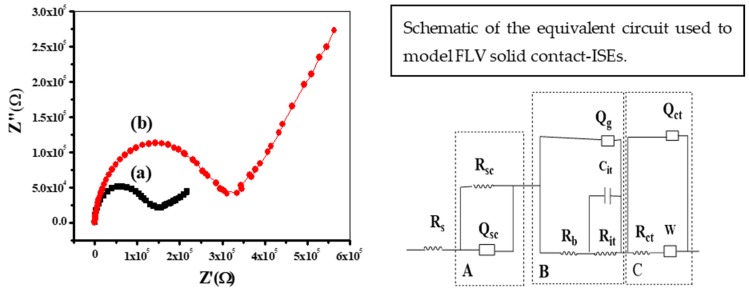
Electrochemical Impedance Spectroscopy (EIS) plots of the applied sensor (**a**) with and (**b**) without MWCNT as a solid contact material.

Electrochemical impedance spectroscopy (EIS) was applied for the proposed sensors in the absence and presence of MWCNTs. The spectra of impedance were recorded at the open-circuit potential in 0.01 V FLV solutions with excitation amplitude of 10 mV. The frequency range starts from 100 kHz to 0.1 Hz. The Nyquist plots (complex plane plots of –Z’’ vs. Z’) on the equivalent circuit models are shown in Figure 8. The bulk impedance of the membrane (*R*_b_) and geometric capacitance (C_g_) can be estimated from the high-frequency semicircle. The values of (*R*_b_) and (*Q*_g_) were found to be (*R*_b_ = 0.15 ± 0.04 MΩ, *Q*_g_ = 30.9 ± 2.4pF) and (*R*_b_ = 0.32 ± 0.03 MΩ, *Q_g_* = 14.7 ± 1.7pF) of the proposed sensors in presence and absence of MWCNTs, respectively. From the low-frequency branch (semicircle), which is characteristic of the type of solid contact used, the double layer capacitance (*Q*sc) was estimated. The low-frequency capacitance (*Q*sc) of the sensors in the presence and absence of MWCNTs was 30.6 ± 1.2 and 5.8 ± 0.9 µF, respectively. These results confirm the Nano-structured features of MWCNTs generate a large double-layer capacitance that revealed enhanced potential stability of the proposed sensors.

All the results arising from EIS and CP measurements confirm that high conductivity and enhanced potential stability of the sensors in the presence of MWCNTs.

The equivalent circuit model that we used to model the experimental data is shown in Figure 8. The circuit comprises 3 parallel blocks labeled A, B and C, and the bulk solution resistance *R*s. Block A represents the solid contact layer where the charge transfer resistance, *R*sc, and the double layer capacitance, *Q*sc, associated with the MWCNTs, are connected in parallel. Block B represents the water uptake occurring over time within the polymeric membrane. Block C describes the diffusion mass transfer with a Warburg element connected to the charge transfer resistance, *R*ct, and the double layer capacity, *Q*_dl_. These last two elements describe the interface between the PVC membrane and the solution.

### 3.4. Analytical Applications

As shown in Table 3, the average recoveries were obtained varied between 98.8–101.9%. The results obtained from the proposed potentiometric method are comparable to the method of HPLC from British pharmacopeia, 2009 [12]. From *t*-student and *F*-tests, the results emphasize that there are no significant differences between the results of two methods and revealed the applicability of the proposed sensor as a new analytical method for FLV determination.

## 4. Conclusions

Herein, a new applicable potentiometric sensor of FLV based on molecular imprinting polymer (MIP) ionophore was developed. MIP was deposited by drop-casting on screen-printed on an alumina substrate. AAm and EGDMA were used as functional monomer and cross-linker with FLV as a template. The new proposed sensor showed high sensitivity with a detection limit of 4.8 × 10^−6^ mol/L with the near-Nernstian cationic slope of 55.0 ± 0.8 mV/decade (*r^2^* = 0.999) and excellent selectivity in the presence of different species. FT-IR and SEM were used to show the characteristic peaks of the imprinting process and the morphologies of the surface of polymeric beads, respectively. MWCNT was used for developing chronopotentiometry and electromagnetic impedance segments, which decreases the potential drift, resistance (R) and increases potential stability, double layer and geometric capacitances. In addition, the utility of the proposed sensor for the determination of FLV in different pharmaceutical and biological samples that exhibited high recoveries and confirmed the validity of the proposed sensor for FLV determination in different samples.

## Figures and Tables

**Table 1:** The potentiometric response of FLV membranes sensor.

Type of Sensor	MWCNT	MIP (mg)	NIP (mg)	PVC (mg)	TPB (mg)	DOP (mg)	THF (mL)	Slope (mV/decade)	Detection Limit (mol/L)	*r* ^2^
**I**	-	12.0	-	68.0	2.0	118.0	2.0	55.2 ± 0.4	4.7 × 10^−6^	0.999
**II**	-	-	-	68.0	2.0	118.0	2.0	55.4 ± 0.5	1.2 × 10^−5^	0.999
**III**	-	12.0	-	68.0	-	118.0	2.0	32.8 ± 1.4	1.0 × 10^−5^	0.995
**IV**	presence	12.0	-	68.0	2.0	118.0	2.0	56.2 ± 0.6	4.3 × 10^−6^	0.999
**V**	-	-	12.0	68.0	-	118.0	2.0	29.7 ± 1.4	1.1 × 10^−5^	0.999

**Table 2:** The selectivity coefficients ($\log\;\log\;K_{\mathrm{FLV},J}^{\mathrm{Pot}}$ ) of the proposed sensor with and without MWCNT.

Interfering Ion	$\log\;K_{\mathrm{FLV},J}^{\mathrm{Pot}}$	
	With MWCNT	Without MWCNT
**K^+^**	−3.41	−3.95
**Na^+^**	−3.74	−3.97
**Ba^2+^**	−3.62	−3.84
**Ca^2+^**	−3.44	−3.83
**Arginine**	−3.85	−4.22
**Caffeine**	−3.84	−3.48
**Glucose**	−3.72	−3.87
**Lactose**	−3.71	−3.84
**Paracetamol**	−3.60	−3.65
**Fluxatine,**	−3.12	−3.11
**Venlafaxine,**	−2.94	−3.01
**Ephedrine**	−3.66	−3.67

**Table 3:** FLV determination in pharmaceutical preparations using the proposed membrane sensor.

Pharmaceutical Product and Source	Nominal Content is Taken, mg tablet^−1^	Found, mg tablet^−1^				*t*-Student Test ^b^	*F*-Test ^b^
		Proposed Method	Mean ^a^ (%) ± SD	Reference Method [12]	Mean ^a^ (%) ± SD		
**Fluxamine tablets (Pharaonia pharmaceuticals “Pharo Pharma”, Egypt)**	50	50.9	101.9 ± 0.8	50.1	100.1 ± 1.3	1.7	5.1
	100	99.1	99.1 ± 0.9	99.9	99.9 ± 1.3	1.1	3.6
**Faverin^®^ tablets (Abbott Healthcare SAS)**	50	49.4	98.8 ± 0.9	50.1	100.1 ± 1.5	1.4	5.5
	100	100.3	100.3 ± 0.9	99.9	99.9 ± 1.4	0.2	5.2

^a^ Mean of three replicate measurements ± standard deviation (SD); ^b^ *t*-Student and *F*-test at 95% confidence level values are 4.30, 19.00 respectively.

## References

[1] Ahmed R.F., Abdel-Rahman R.F., Farid O.A., El-Marasy S.A., Hessin A.F. (2014). Combined hepatoprotective and antidepressant effects of resveratrol in an acute model of depression. Bull. Fac. Pharm. Cairo Uni..

[2] Serafini G. (2012). Neuroplasticity and major depression, the role of modern antidepressant drugs. World J. Psychiatry.

[3] Dsm-Iv-tr A. (2000). Diagnostic and Statistical Manual of Mental Disorders, Text Revision.

[4] El Tanbouly N., El Sayed A.M., Ali Z.Y., Abdel Wahab S., El Gayed S.H., Ezzat S.M., Senousy E., Safwat A., Choucry M.A., Abdel-Sattar E. (2017). Antidepressant-Like Effect of Selected Egyptian Cultivars of Flaxseed Oil on a Rodent Model of Postpartum Depression. Evid-Based Compl Alt..

[5] Nomura S. (2004). Characteristics and use of new antidepressant drugs. Japan Med. Assoc. J..

[6] Katzung B. (2007). Drugs of Abuse, Basic and Clinical Pharmacology.

[7] Wilde M.I., Plosker G.L., Benfield P. (1993). Fluvoxamine. Drugs.

[8] Pullen R.H., Fatmi A.A. (1992). Determination of fluvoxamine in human plasma by high-performance liquid chromatography with fluorescence detection. J. Chromatogr. B Biomed..

[9] De Jong G. (1980). The use of a pre-column for the direct high-performance liquid chromatographic determination of the anti-depressants clovoxamine and fluvoxamine in plasma. J. Chromatogr. B: Biomed. Sci. Appl..

[10] Wong S.H., Kranzler H.R., Dellafera S., Fernandes R. (1994). Determination of fluvoxamine concentration in plasma by reversed-phase liquid chromatography. Biomed. Chromatogr..

[11] Ulu S.T. (2006). Determination and validation of an LC method for fluvoxamine in tablets. Chromatographia.

[12] Comission B.P. (2009). British Pharmacopoeia.

[13] U.P (2007). USP30-NF25.

[14] Härtter S., Wetzel H., Hiemke C. (1992). Automated determination of fluvoxamine in plasma by column-switching high-performance liquid chromatography. Clin. Chem..

[15] Ohkubo T., Shimoyama R., Otani K., Yoshid K., Higuchi H., Shimizu T. (2003). High-performance liquid chromatographic determination of fluvoxamine and fluvoxamino acid in human plasma. Anal. Sci..

[16] Foglia J.P., Birder L.A., Perel J.M. (1989). Determination of fluvoxamine in human plasma by high-performance liquid chromatography with ultraviolet detection. J. Chromatogr. B Biomed..

[17] Wille S.M., Van Hee P., Neels H.M., Van Peteghem C.H., Lambert W.E. (2007). Comparison of electron and chemical ionization modes by validation of a quantitative gas chromatographic–mass spectrometric assay of new generation antidepressants and their active metabolites in plasma. J. Chromatogr. A.

[18] Berzas Nevado J., Villasenor Llerena M., Contento Salcedo A., Nuevo E.A. (2000). Determination of fluoxetine, fluvoxamine, and clomipramine in pharmaceutical formulations by capillary gas chromatography. J. Chromatogr. Sci..

[19] Nevado J.B., Salcedo A.C., Llerena M.V., Nuevo E.A. (2000). Method development and validation for the simultaneous determination of fluoxetine and fluvoxamine in pharmaceutical preparations by capillary electrophoresis. Anal. Chim..

[20] Flores J.R., Nevado J.J.B., Salcedo A.M.C., Díaz M.P.C. (2004). Development of a capillary zone electrophoretic method to determine six antidepressants in their pharmaceutical preparations. Experimental design for evaluating the ruggedness of method. J. Sep. Sci..

[21] Lin E.P., Chiu T.C., Hsieh V. (2016). Dispersive liquid–liquid microextraction combined with acetonitrile stacking through capillary electrophoresis for the determination of three selective serotonin reuptake inhibitor drugs in body fluids. J. Sep. Sci..

[22] Darwish I.A., Amer S.M., Abdine H.H., Al-Rayes L.I. (2009). Spectrofluorimetric determination of fluvoxamine in dosage forms and plasma via derivatization with 4-chloro-7-nitrobenzo-2-oxa-1, 3-diazole. J. Fluoresc..

[23] Yang D., He Y., Chen F. (2017). Determination of fluvoxamine maleate in human urine and human serum using alkaline KMnO4–rhodamine B chemiluminescence. Luminescence.

[24] Hassanzadeh J., Amjadi M. (2015). Sensitive and selective determination of fluvoxamine maleate using a sensitive chemiluminescence system based on the alkaline permanganate–Rhodamine B–gold nanoparticles reaction. Luminescence.

[25] Ajayi R.F., Nxusani E., Douman S.F., Jonnas A., Baker P.G.L., Iwuoha E.I. (2016). An amperometric cytochrome P450-2D6 biosensor system for the detection of the selective serotonin reuptake inhibitors (SSRIs) paroxetine and fluvoxamine. J. Nano Res.-Sw..

[26] Nevado J.B., Rodriguez Flores J., Castañeda Peñalvo G. (2000). Voltammetric Behavior of Fluvoxamine Using Square-Wave and Adsorptive Stripping Square-Wave Techniques. Determination in Pharmaceutical Products. Electroanalysis.

[27] Elmali F., Alpdogan G., Sungur S., Aycan Ş. (2000). Polarographic determination of fluvoxamine maleate in tablets. Turk. J. Chem..

[28] Alhaider A.A., Hagga M.E.M., Alawady M.E., Gad-kariem E.A. (1993). Spectrophotometric Determination of Fluvoxamine in Tablets Based on Charge-Transfer Complex with Chloranil. Anal. Lett..

[29] Kishore M., Koteswarao A., Janardhan M. (2011). Validation of new spectrophotometric methods for the determination of Fluvoxamine as maleate in pharmaceutical formulations. Res. J. Pharm. Tech..

[30] Devarajan S., Manavalan G., Balasubramanian K., Annamalai J., Madduri N. (2015). A new spectrophotometric method for the determination of fluvoxamine maleate in pure form and in pharmaceutical formulation. J. Appl. Pharm. Sci..

[31] Hassan S.S.M., Amr A.E.G.E., El-Naby H.A., El-Naggar M., Kamel A.H., Khalifa N.M. (2019). Novel aminoacridine sensors based on molecularly imprinted hybrid polymeric membranes for static and hydrodynamic drug quality control monitoring. Materials.

[32] Kamel A.H., Soror T.Y., Al-Romian F.M. (2012). Flow through potentiometric sensors based on molecularly imprinted polymers for selective monitoring of mepiquat residue, a quaternary ammonium herbicide. Anal. Methods.

[33] El-Kosasy A., Kamel A.H., Hussin L., Ayad M.F., Fares N. (2018). Mimicking new receptors based on molecular imprinting and their application to potentiometric assessment of 2, 4-dichlorophenol as a food Taint. Food Chem..

[34] El-Naby E.H., Kamel A.H. (2015). Potential transducers based man-tailored biomimetic sensors for selective recognition of dextromethorphan as an antitussive drug. Mat. Sci. Eng. C.

[35] Elbehery N.H.A., Amr A.E.G.E., Kamel A.H., Elsayed E.A., Hassan S.S.M. (2019). Novel potentiometric 2, 6-Dichlorophenolindophenolate (DCPIP) membrane-based sensors: Assessment of their input in the determination of total phenolics and ascorbic acid in beverages. Sensors.

[36] Abdalla N.S., Amr A.E.G.E., El-Tantawy A.S.M., Al-Omar M.A., Kamel A.H., Khalifa N.M. (2019). Tailor-made specific recognition of cyromazine pesticide integrated in a potentiometric strip cell for environmental and food analysis. Polymers.

[37] Kamel A.H., Hassan A.M.E. (2016). Solid contact potentiometric sensors based on host-tailored molecularly imprinted polymers for creatine assessment. Int. J. Electrochem..

[38] Mirata F., Resmini M. (2015). Molecularly Imprinted Polymers for Catalysis and Synthesis, Molecularly Imprinted Polymers in Biotechnology.

[39] Lok C., Son R. (2009). Application of molecularly imprinted polymers in food sample analysis–a perspective. Int. Food Res. J..

[40] Chen G., Jin M., Du P., Zhang C., Cui X., Zhang Y., She Y., Shao H., Jin F., Wang S. (2017). A sensitive chemiluminescence enzyme immunoassay based on molecularly imprinted polymers solid-phase extraction of parathion. Anal. Biochem..

[41] Azodi-Deilami S., Abdouss M., Kordestani D. (2014). Synthesis and characterization of the magnetic molecularly imprinted polymer nanoparticles using N, N-bis methacryloyl ethylenediamine as a new cross-linking agent for controlled release of meloxicam. Appl. Biochem. Biotechnol..

[42] Asadi E., Azodi-Deilami S., Abdouss M., Khaghani S. (2012). Cyproterone synthesis, recognition and controlled release by molecularly imprinted nanoparticle. Appl. Biochem. Biotechnol..

[43] Haupt K. (2001). Molecularly imprinted polymers in analytical chemistry. Analyst.

[44] Ye L., Haupt K. (2004). Molecularly imprinted polymers as antibody and receptor mimics for assays, sensors and drug discovery. Anal. Bioanal. Chem..

[45] Li H.-H., Wang H.-H., Li W.-T., Fang X.-X., Guo X.-C., Zhou W.-H., Cao X., Kou D.-X., Zhou Z.-J., Wu S.-X. (2016). A novel electrochemical sensor for epinephrine based on three dimensional molecularly imprinted polymer arrays. Sens. Actuators B Chem..

[46] Chen H., Zhang W., Yang N., Chen C., Zhang M. (2018). Chitosan-Based Surface Molecularly Imprinted Polymer Microspheres for Sustained Release of Sinomenine Hydrochloride in Aqueous Media. Appl. Biochem. Biotechnol..

[47] Vasapollo G., Sole R.D., Mergola L., Lazzoi M.R., Scardino A., Scorrano S., Mele G. (2011). Molecularly imprinted polymers: Present and future prospective. Int. J. Mol. Sci..

[48] Galal Eldin A., Amr E., El-Galil A., Kamel A.H., Hassan S.S.M. (2019). Screen-printed Microsensors Using Polyoctyl-thiophene (POT) Conducting Polymer As Solid Transducer for Ultratrace Determination of Azides. Molecules.

[49] Galichet L., Moffat A., Osselton M., Widdop B. (2004). Clarke’s Analysis of Drugs and Poisons.

[50] Zhang W., She X., Wang L., Fan H., Zhou Q., Huang X., Tang J. (2017). Preparation, characterization and application of a molecularly imprinted polymer for selective recognition of sulpiride. Materials.

[51] Alizadeh T., Ganjali M.R., Akhoundian M. (2012). Synthesis and application of different nano-sized imprinted polymers for the preparation of promethazine membrane electrodes and comparison of their efficiencies. Int. J. Electrochem. Sci..

[52] Ratautaite V., Plausinaitis D., Baleviciute I., Mikoliunaite L., Ramanaviciene A., Ramanavicius A. (2015). Characterization of caffeine-imprinted polypyrrole by a quartz crystal microbalance and electrochemical impedance spectroscopy. Sens. Actuators B.

[53] Baleviciute I., Ratautaite V., Ramanaviciene A., Balevicius Z., Broeders J., Crouxd D., McDonaldd M., Vahidpourd F., Thoelend R., De Ceuninckd W. (2015). Evaluation of theophylline imprinted polypyrrole film. Synth. Met..

[54] Bakker E. (1997). Determination of Unbiased Selectivity Coefficients of Neutral Carrier-Based Cation-Selective Electrodes. Anal. Chem..

